# Melatonin: Roles and Molecular Mechanisms in Metabolic Dysfunction-Associated Steatotic Liver Disease

**DOI:** 10.3390/biom16091357

**Published:** 2026-09-17

**Authors:** Binjing Pan, Xiaoyu Lv, Xinyuan Guo, Jingfang Liu

**Affiliations:** 1 The First Clinical Medical College, Lanzhou University, Lanzhou 730000, China; 2 Department of Endocrinology, The First Hospital of Lanzhou University, Lanzhou 730000, China

**Keywords:** metabolic dysfunction-associated steatotic liver disease, melatonin, oxidative stress

## Abstract

Metabolic dysfunction-associated steatotic liver disease (MASLD) is a common metabolic disorder that affects the endocrine and digestive systems. It often leads to multi-system metabolic abnormalities, including disturbances in blood glucose and lipid levels. MASLD ranges from normal liver to steatosis, followed by fibrosis and cirrhosis, eventually leading to hepatocellular carcinoma. This disease can exacerbate liver-related complications and promote the development of extrahepatic diseases in other systems, including the cardiovascular and renal systems. Melatonin, a regulator of neurotransmitters, hormones, and biological responses, modulates oxidative stress, buffers free radicals, influences mitochondrial function, and regulates energy. Therefore, melatonin may serve as a novel adjunctive therapeutic agent for delaying or inhibiting the development of MASLD. We summarize the recent research progress on the effects of melatonin on MASLD and related molecular mechanisms, and identify new ideas and targets for MASLD treatment.

## 1. Introduction

Metabolic dysfunction-associated steatotic liver disease (MASLD) is the most common chronic liver disorder and is characterized by metabolic dysfunction. Due to lifestyle changes, its global prevalence rate can reach 38% [1]. As blood glucose and lipid disorders worsen, the prevalence of diseases such as obesity and overweight increases. MASLD is a systemic metabolic disorder characterized by severe liver and extrahepatic complications [1]. Studies indicate that approximately 1/3 of patients with fatty liver disease are expected to progress to metabolic dysfunction-associated steatohepatitis (MASH) and advanced liver disease [1]. Over the past two decades, the prevalence of MASLD among Chinese adults has been approximately 29.6%, with a higher incidence in men (34.8%) than in women (23.5%). MASLD is a chronic disease of global concern. Owing to the lack of effective diagnostic and therapeutic tools, the clinical management of MASLD faces serious challenges, imposing a heavy economic and social burden [2].

According to the 2023 multi-society Delphi consensus, the diagnosis of MASLD requires the presence of hepatic steatosis together with at least one cardiometabolic risk factor, such as obesity, type 2 diabetes, or dyslipidemia. This nomenclature replaces the previous term nonalcoholic fatty liver disease (NAFLD) and reflects the central role of metabolic dysfunction in disease pathogenesis. Lifestyle modification remains the cornerstone of management [3]. However, recent advances have expanded pharmacological options. In 2024, resmetirom, a thyroid hormone receptor-beta agonist, became the first agent approved for the treatment of MASH with moderate to advanced fibrosis. Other therapeutic approaches, including glucagon-like peptide-1 receptor agonists and peroxisome proliferator-activated receptor agonists, are currently under investigation and have shown promising results in phase 2 and 3 trials [4]. These developments highlight the need to evaluate adjunctive therapies, such as MT, within the context of an evolving treatment landscape.

In this review, we use the term MASLD, which was introduced by a multi-society Delphi consensus in 2023 to replace the previous terminology of NAFLD. Similarly, the term MASH replaces nonalcoholic steatohepatitis (NASH). Where older studies used the previous nomenclature, we have retained the original terminology to accurately reflect the study design and diagnostic criteria of that time.

MT was discovered in bovine pineal gland extracts, and its secretion follows a circadian rhythm, peaking during the dark period [5]. Studies have shown that MT is synthesized not only in the pineal gland but also in various tissues, including blood vessels, the gastrointestinal tract, and lymphocytes [6]. Besides regulating sleep, mood, aging, and circadian rhythms, MT also exhibits many other important functions, such as regulating immune responses, eliminating free radicals, regulating the intestinal microbiota, stabilizing mitochondrial structure and function, and influencing mutations and carcinogenesis [5]. MT influences tissue and organ functions by regulating oxidative stress and sleep states. Research indicates that it can act as an antioxidant in cardiac tissue to slow the progression of heart disease [7].

Studies have shown that MT can improve lipid metabolism disorders and is associated with the occurrence and progression of MASLD. This article reviews recent research progress on the effects of MT on MASLD, providing a new idea and target for the pathogenesis and clinical diagnosis of MASLD patients in clinical practice.

## 2. Biosynthesis and Physiological Function of MT

MT (N-acetyl-5-methoxytryptophan) is a hormone related to regulating biological rhythms. This hormone is secreted by the pineal gland at night and exhibits a circadian rhythm. The pineal gland weighs approximately 100–150 mg and is rich in blood vessels [8]. The concentration of MT during the day is 0–20 pg/mL, while it can reach up to 100 pg/mL at night. In addition to the pineal gland, which was first to be discovered, other parts and tissues of the body can also produce MT. However, the MT produced in these areas can only exert local regulatory effects. For example, the concentration of MT in the gastrointestinal tract is approximately 100 times that in the blood [8].

The regulation of circadian rhythms was the earliest identified physiological function of MT. The production of MT is inhibited by light and increases in the absence of light. The biological rhythmic effect of MT can regulate tissues, making them consistent with the circadian cycle, thereby influencing the metabolic level of the body [9]. The circadian rhythm controls various behavioral processes, such as feeding and fasting, wakefulness, and sleep, in a 24 h cycle to ensure that physiological behaviors are synchronized with the daily environmental pattern and optimize energy balance. Continuous exposure to light and changes in diet-induced circadian rhythm disorders can damage energy balance and promote the development of metabolic disorders. MT is a potent natural modulator of the circadian rhythms involved in glucose and lipid metabolism [10].

Studies have shown that MT can reduce oxidative stress and has significant antioxidant effects. It promotes the production of antioxidants, scavenges oxygen free radicals, repairs damaged DNA, and enhances the expression of normal molecules [9]. MT interacts with copper and iron to remove them from the intracellular and extracellular environments in the form of metal-ion complexes, thereby reducing metal-dependent free radical generation and minimizing damage to tissues and organs. Studies have indicated that MT competitively binds to iron, thereby inhibiting ferroptosis [9]. MT can stimulate the production of antioxidant enzymes, thereby relieving the toxicity of strong oxidants. Under conditions of high oxidative stress, MT maintains enzyme activity and protects proteins from damage by free radicals, thereby enhancing the level of glutathione (GSH) within the cell [11].

MT has been shown to enhance antitumor immune responses by directly or indirectly regulating NK and T cells. MT modulates the immune response and enhances the release of IL-1 by lymphocytes and T cells [12]. Endogenous synthesis of MT occurs in the immune system and is considered another source of MT, apart from the pineal gland. Moreover, melatonin receptors have been demonstrated to be distributed in immune tissues and cells. Under normal and immunosuppressive conditions, MT exerts an immune-enhancing effect by increasing the number of immune-related cells. However, in cases of exacerbated immune responses, MT also reduces immune responses, manifested as reduced infiltration of neutrophils and migration of neutrophils and monocytes. MT regulates the phagocytic function of immune cells, thereby exerting an immune-modulating effect [13]. After the administration of MT to normal and immunosuppressed mice, the production of T helper cells increased, and the production of CD8+ T cells decreased [14].

**Figure 1:**
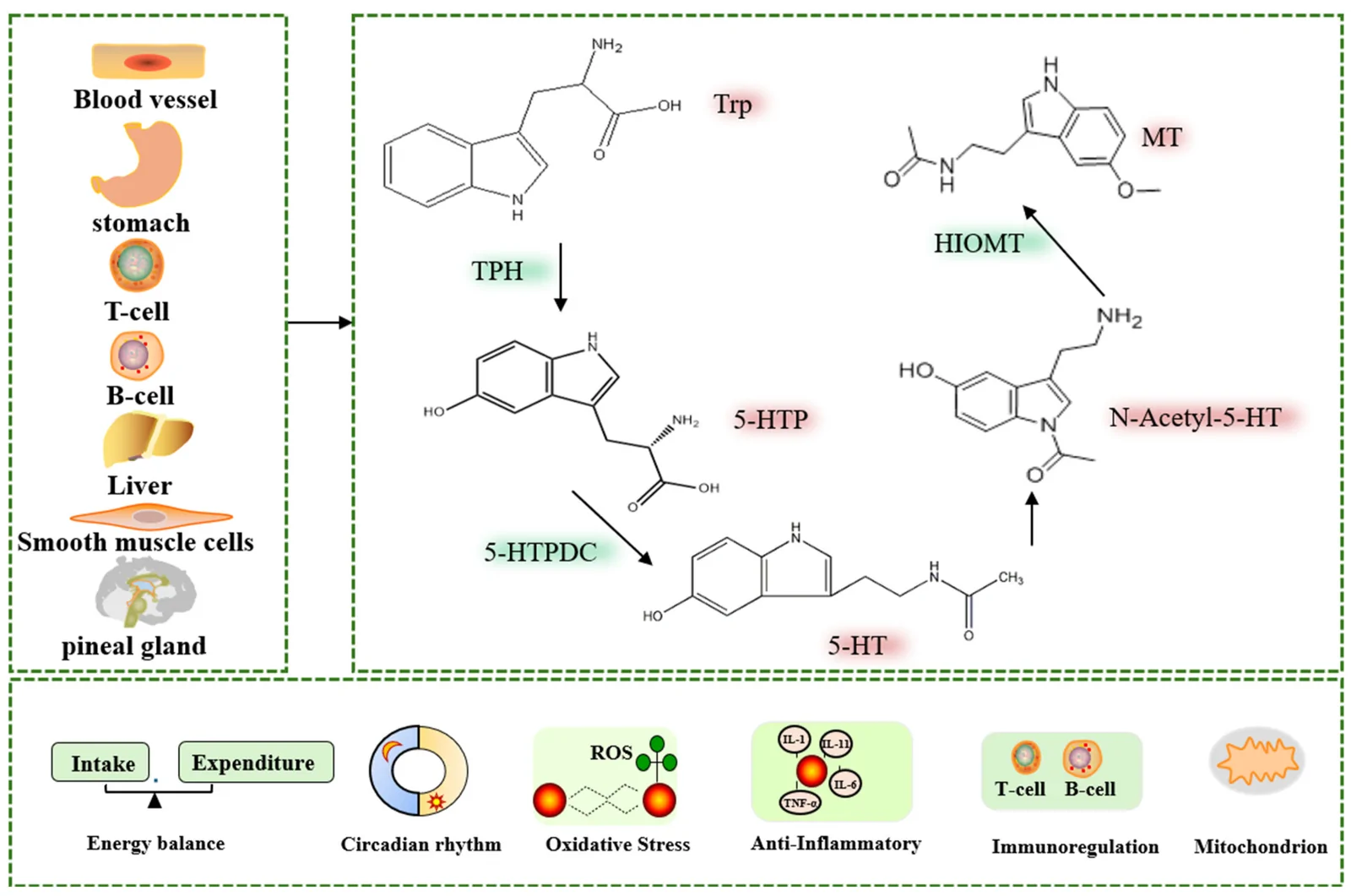
The biosynthesis and physiological functions of MT. Note: MT: melatonin; Trp: Tryptophan; TPH: tryptophan hydroxylase; 5-HTPDC: 5-Hydroxytryptophan decarboxylase; 5-HTP: 5-hydroxytryptophan; ROS: reactive oxygen species; 5-HT: 5-Hydroxytryptamine; N-Acetyl-5-HT: N-acetyl-5-hydroxytryptamine; HIOMT: methyltransferase; IL-6: Interleukin-6; IL-1: Interleukin-1; IL-11: Interleukin-11.

MT is released in a circadian rhythm, whereas other hormones involved in the regulation of energy homeostasis, such as insulin and leptin, follow a circadian pattern of secretion and release. This indicates that they are closely related to mitochondrial bioenergetics [14]. Leptin, an adipokine primarily secreted by white adipose tissue, transmits nutritional reserve signals to the central nervous system, thereby suppressing appetite, reducing food intake, and promoting energy breakdown and utilization. Insufficient leptin levels in the body can lead to uncontrolled appetite and disrupt glucose and lipid metabolism in the liver. MT helps regulate mitochondrial homeostasis, improves mitochondrial function, and removes free radicals [15]. Mitochondrial ROS are mainly produced in the mitochondria, but are also cleared by antioxidant enzymes within the mitochondria to maintain the redox balance. A decline in antioxidant capacity or accumulation of reactive oxygen species (ROS) is closely related to mitochondrial dysfunction. MT maintains mitochondrial structural integrity and functional homeostasis through various mechanisms, including the protection of mitochondrial DNA (mtDNA), activation of uncoupling proteins, and inhibition of the mitochondrial permeability transition pore (mPTP), ultimately preserving optimal mitochondrial membrane potential. MT acts as an indirect antioxidant to prevent cytoplasmic calcium overload. These effects are crucial for reducing oxidative stress, thereby reducing mtDNA damage and its release into the cytoplasm [16]. Mitochondrial autophagy effectively prevents the accumulation of dysfunctional mitochondria by actively clearing damaged organelles, thereby maintaining mitochondrial homeostasis. Therefore, inappropriate mitochondrial autophagy (excessive or insufficient autophagy) can impair mitochondrial function, leading to the disruption of cellular energy metabolism. MT promotes the expression of Sirtuin 1 (SIRT1) and alleviates lipopolysaccharide-induced excessive mitochondrial autophagy, thereby improving mitochondrial function [17]. The biosynthesis and physiological functions of MT are shown in Figure 1.

## 3. The Pathophysiologic Mechanisms of MASLD

MASLD is a multifactorial disease whose core pathogenesis involves gene–environment interactions that lead to systemic metabolic dysfunction, resulting in the disruption of intercellular molecular pathways and functional disorders in hepatocytes, hepatic sinusoidal endothelial cells, stromal cells, and recruited immune cells [18].

Patients with MASLD exhibit significant insulin resistance (IR). IR promotes efflux of free fatty acids (FFAs) from adipocytes. These fatty acids enter the liver and cause abnormal lipid deposition. Additionally, patients with MASLD often exhibit an increase in de novo lipogenesis, resulting in an excessive supply of FFAs to the hepatocytes [19]. Increased FFA supply induces lipotoxic stress. Cells metabolize excess fatty acids via two primary pathways. They can either directly break down energy via mitochondrial beta-oxidation or convert fatty acids into triglycerides through esterification in hepatocytes. These triglycerides are then assembled into very low-density lipoproteins (VLDL-C), released into the bloodstream, and subsequently cleared [19]. When the subcutaneous fat tissue content exceeds the normal level, fat may accumulate ectopically in the visceral region, leading to an increased release of FFAs into the liver, accompanied by the activation of proinflammatory cytokines and adipokines [18]. Excessive accumulation of FFAs in the liver tissue and cells results in a series of harmful consequences, including mitochondrial dysfunction, metabolic disorders, activation of inflammation-related signaling pathways, exacerbation of inflammatory responses, and promotion of oxidative stress. This is considered the initial driving factor in the transition from simple steatosis to MASH [18].

Mitochondria are sites of oxidative metabolism in eukaryotic organisms and play a vital role in energy metabolism, mainly through oxidative phosphorylation to produce adenosine triphosphate (ATP), which provides a continuous energy supply for cells [20]. The integrity of this function is crucial for maintaining hepatic metabolic homeostasis, and its disruption is closely associated with hepatic metabolic dysfunction [20]. Mitochondrial dysfunction leads to excessive production of ROS, which in turn causes oxidative damage to major biomolecules within cells, including proteins, lipids, and DNA. The metabolites produced by the tricarboxylic acid cycle are essential for biosynthesis. They form the core of energy metabolism and participate in the regulation of vital biological processes, such as cell growth, differentiation, and apoptosis. The disruption of mitochondrial structure and function can promote the progression of liver disease through multiple mechanisms. First, it inhibits the tricarboxylic acid cycle, leading to metabolic disorders [20]. It can also activate hepatic oxidative stress and inflammatory responses by inducing the release of cytokines from adipose tissue, represented by adipokines, Tumor necrosis factor-α (TNF-α), and IL-6, ultimately leading to liver injury and fibrosis [21].

The transformation of Metabolic dysfunction-associated fatty liver (MAFL) to MASH is caused by specific responses and interactions between liver cells and various stimuli. Lipotoxic injury and the secretion of inflammatory cytokines can stimulate oxidative stress reactions in the liver, exacerbating the inflammatory response and leading to the development of liver fibrosis [22]. Liver fat deposition leads to dysregulated apoptosis of hepatocytes, whereas Kupffer cells and adipocytes are activated and secrete inflammatory mediators. In this microenvironment, the stellate cells transform into contractile myofibroblasts. Their core molecular phenotype involves excessive production of type I collagen and abnormal upregulation of extracellular matrix (ECM) proteins such as alpha-smooth muscle actin and fibronectin, which are important factors contributing to the phenotypic heterogeneity of NAFLD [22].

The dysfunction of the gut microbiota plays a significant role in the development of MASLD, and its alterations are closely associated with disease onset. Patients with MASLD have significant metabolic disorders that lead to continuous changes in energy acquisition and decreased microbial diversity [23]. Disruption of both the intestinal epithelial barrier and intestinal vascular barrier in MASH causes the migration of neutrophils in the intestine and an increase in bacterial translocation in the circulation [23]. When these products bind to the liver cells, they may trigger an inflammatory response.

Dysbiosis, including an increase in Proteobacteria and Enterobacteriaceae and a decrease in the phylum Firmicutes, leads to an increase in harmful microbial metabolites. Dysbiosis of the gut microbiota and its associated microbial metabolites can directly or indirectly exacerbate liver lesions through mechanisms such as the gut–liver axis and inflammatory signaling pathways, thereby promoting the progression of liver disease from steatosis to fibrosis [23]. Moreover, microorganisms can affect the release of incretin hormones, which may indirectly affect glucose metabolism [23].

Bile acids are also associated with the progression of MAFLD. Dysfunction of the gut–liver axis can trigger abnormalities in bile acid metabolism. This primarily involves the disruption of hepatic bile acid synthesis and the inhibition of dihydroxylation performed by the gut microbiota. This leads to an imbalance in bile acid homeostasis, which exacerbates metabolic disorders [24]. The gut microbiota is also associated with alcohol consumption. Alcohol is hepatotoxic and alters intestinal permeability, thereby inducing endotoxemia. Endotoxins, also known as lipopolysaccharides, are key components of bacterial cell walls. Endotoxins produced by gut microbiota can directly affect the liver via the portal venous system and exacerbate liver lesions. Increased intestinal permeability triggers endotoxemia, which, in turn, induces inflammatory responses through cytokines. Kupffer cells in the liver can be activated by endotoxins, thereby accelerating the progression of liver fibrosis [25].

Patients with chronic liver disease often experience multiple sleep disorders. Patients with MASLD experience various forms of sleep disorders, with the most common types being obstructive sleep apnea, insomnia, and restless legs syndrome [26]. Leptin exists in two forms in the circulatory system: free leptin and protein-bound leptin. This hormone, which is primarily synthesized by the white adipose tissue, is a key component of energy homeostasis and neuroendocrine regulation. The secretion of leptin by adipocytes is strictly regulated by the circadian rhythm. Disruption of circadian rhythms not only leads to abnormal leptin secretion but may also promote the progression of MASLD by disturbing energy metabolism homeostasis [27]. Circadian rhythm disruption caused by environmental and behavioral factors leads to decreased serum leptin levels. These hormonal changes are associated with an increased incidence of obesity and diabetes. Disruption of the circadian clock leads to leptin resistance in the hypothalamus and endoplasmic reticulum stress, resulting in abnormal lipid metabolism [27]. This is a period of increased energy storage. During this period, the liver promotes the synthesis of carbohydrates such as glycogen, while adipose tissue serves as the primary site for storing glycogen and lipids. Nighttime is a sleep period, during which parts of the above-mentioned body reserves are mobilized. Disruption of the circadian rhythm has an adverse effect on energy balance and increases metabolic risk factors [28].

## 4. The Influence of MT on MASLD and Its Molecular Mechanism

### 4.1. Antioxidant Stress

MT exerts direct and indirect antioxidant effects [29]. It can directly eliminate ROS and reactive nitrogen species (RNS) through non-receptor mechanisms and capture free radicals through two pathways that rely on single-electron transfer and hydrogen transfer. MT has also been found to directly scavenge nitrite anions and hypochlorous acid. MT is a key endogenous molecule that scavenges oxygen- and nitrogen-free radicals. It can induce detoxification at both physiological and pharmacological concentrations [30]. MT modulates the expression of superoxide dismutase (SOD) and glutathione peroxidase (GPx) mRNA by regulating their transcription under oxidative stress, thereby influencing their corresponding enzyme activities. Low concentrations of MT exert antioxidant effects by regulating GPx, whereas high concentrations of MT have direct free radical-scavenging effects [30]. MT are lipophilic and can rapidly reach subcellular compartments, including the mitochondria. MT acts directly on the electron transport chain (ETC) through its high reduction potential, enhancing electron flow to promote ATP production. However, this process also generates ROS/RNS. High concentrations of nitric oxide (NO) significantly inhibit the ETC and exacerbate inflammatory responses. Under oxidative stress, MT primarily exerts mitochondrial protective effects by upregulating reduced GSH levels and downregulating oxidized glutathione (GSSG) and hydrogen peroxide [31].

Studies have shown that MT acting on HepG2 cells treated with high fatty acids increases GSH and SOD levels and decreases lipid ROS and malondialdehyde (MDA) levels. The same conclusion was reached in animal experiments. MT antagonizes abnormal lipid synthesis and peroxidation within the liver, thereby reducing oxidative stress and protecting the liver from damage [32]. MT exerts antioxidant effects through multiple mechanisms, including direct scavenging of oxygen free radicals, upregulation of endogenous antioxidant enzyme activity, enhancement of mitochondrial oxidative phosphorylation efficiency to reduce electron leakage, and stimulation of other antioxidant secretion [33].

### 4.2. Anti-Inflammatory

Persistent chronic low-grade inflammation can lead to increased oxidative stress and dysfunction of the vascular endothelium in the liver. Excessive deposition of the extracellular matrix and collagen is the primary pathological manifestation of liver fibrosis, forming the basis for progression to cirrhosis and HCC [34]. In MASH, the dysfunction of hepatic sinusoidal endothelial cells manifests as impaired autophagy capacity, increased expression of proinflammatory and adhesion molecules, and exacerbated hepatic inflammatory responses. This is a key mechanism that promotes hepatic endothelium-to-mesenchymal transition and fibrosis development. Normal phenotype and function of hepatic sinusoidal endothelial cells inhibit MASH fibrosis progression. As gatekeepers of the hepatic microenvironment, capillarization of these cells is a critical step in disease, and abnormal accumulation of the extracellular matrix is the direct pathological basis for progressive fibrosis [35].

MT can improve some phenotypes of MASLD, including liver lipid accumulation, fatty hepatitis, and liver fibrosis [36,37]. MT regulates pro-inflammatory cytokines through the Nuclear Factor kappa-light-chain-enhancer of activated B cells (NF-κB)/NOD-like receptor family pyrin domain containing 3(NLRP3) pathway. MT can effectively block the activation of the NLRP3 inflammasome, thereby inhibiting the formation of caspase-1 and IL-1β. Additionally, MT effectively prevents excessive production of inflammatory mediators by suppressing cyclooxygenase (COX) expression [37,38]. MT reduces TXNIP expression and inhibits ROS and NLRP3 [39]. MT upregulates antioxidant proteins, such as Nrf2, promoting ROS clearance and elimination [39].

### 4.3. Protect the Structure of Mitochondria

Mitochondria play crucial roles as key organelles in the regulation of hepatocyte homeostasis. In metabolic diseases, such as fatty liver, the most common pathological manifestations include mitochondrial structural and functional impairment, as well as the production of inflammatory factors. These impairments manifest as ultrastructural abnormalities, morphological alterations, reduced respiratory chain activity, impaired ATP synthesis, increased membrane permeability, excessive ROS production, mtDNA deletions induced by oxidative stress, and decreased β-oxidation efficiency [20].

The aggravation of liver fibrosis leads to an increase in aerobic glycolysis. MT regulates glucose homeostasis by acting as an inhibitor of glycolysis and suppressing the Warburg effect [6]. MT directly enhances mitochondrial β-oxidation and promotes the transfer of acetyl-CoA into mitochondria to facilitate fatty acid metabolism [40]. Dysregulation of lipid metabolism leads to the accumulation of toxic lipids and promotes endoplasmic reticulum stress, which is characterized by inflammation, hepatocyte damage, and cell apoptosis. MT enters cells through specific transporters (such as GLUT1, PEPT1/2) and binds to cell membrane receptors (such as MT1R and MT2R), changing the activity of various signaling molecules involved in endoplasmic reticulum stress. MT reduces endoplasmic reticulum stress to maintain homeostasis through various systems such as the ubiquitin–proteasome pathway and UPR [41]. MT regulates mitochondrial energy metabolism, promotes mitochondrial fusion, and protects the mitochondria from damage by modulating autophagy, thereby maintaining normal mitochondrial function. MT increases electron transport chain (ETC) production and enhances oxidative phosphorylation (OXPHOS) [42]. MT also promotes ATP production in rat brain and liver mitochondria by boosting OXPHOS [42,43].

### 4.4. Metabolic Regulation

Patients with MASLD often exhibit varying degrees of IR and abnormal glucose and lipid metabolism. MT not only improves insulin sensitivity and regulates glucose homeostasis, but also regulates lipid metabolism, including fat synthesis, fat breakdown, and mitochondrial biogenesis [44]. MT can improve high-density lipoprotein cholesterol (HDL-C) levels in obese patients, diabetic rats, and diet-induced hypercholesterolemic rats, and reduce triglyceride and low-density lipoprotein levels [44].

MT is closely associated with insulin and may reduce insulin secretion by affecting the cyclic adenosine monophosphate (cAMP) pathway. Additionally, cGMP enhances the function of B cells and acts as a mediator to promote insulin secretion by these cells during the hyperglycemic cycle. MT inhibits the cyclic guanosine monophosphate (cGMP) signaling pathway in pancreatic B cells, leading to reduced insulin secretion. MT improves IR by suppressing c-Jun N-terminal kinase (JNK) activity, enhancing mitochondrial function, and lowering oxidative stress levels [45]. Administration of MT to high-fat diet-fed rats improved the degree of liver steatosis, inflammation, and fibrosis, reduced the insulin resistance index and liver enzyme levels, and increased the level of reduced glutathione [46].

### 4.5. Regulation of Gut Microbiota

The distribution and composition of the gut microbiota can influence hepatic glucose and lipid metabolism, disrupt the expression of inflammatory factors in the liver, and exacerbate conditions, thereby directly promoting the progression from MASLD to MASH fibrosis [47]. MT has been shown to directly influence the metabolism of gut microbiota, affecting the types and abundance of microbial communities within the gut flora, and can improve gut health by reducing oxidative stress, regulating autophagy, and suppressing inflammation, thereby inducing gut microbiota remodeling [48]. In obese humans and mice, the abundance of Bacteroidetes in the gut decreases, whereas the abundance of Firmicutes increases. In mice fed a high-fat diet, MT supplementation reversed this effect [49]. Elevated lipopolysaccharide (LPS) levels in the gut microbiota impair the intestinal barrier function and induce inflammation, leading to increased LPS levels in the bloodstream. This activates the NF-κB signaling pathway and induces the expression of pro-inflammatory cytokines. Experiments demonstrate that MT antagonizes LPS-CD14-TLR4-mediated signaling events in bovine mammary epithelial cells and downregulates the transcription of key inflammatory factors [50].

Inflammasomes, such as 6-containing pyrazine domain protein (NLRP6), are key effector molecules that regulate intestinal permeability and interact with gut bacteria. The balance of intestinal homeostasis and the regulation of intestinal permeability largely depend on the function of these inflammasomes. MT reduces the incidence of sepsis by activating the α7 nicotinic acetylcholine receptor (α7nAChr) and inhibiting the activation of NLRP3 [51]. MT improves Cd-induced liver injury, reshapes the intestinal microbiota, reduces liver bile acid synthesis, enhances intestinal barrier function, and reduces bacterial lipopolysaccharide leakage from the intestine [52]. The activation of FXR (farnesoid X receptor) by MT triggers the activation of transcription factor 4 (ATF4) through trimethylamine-N-oxide (TMAO) signaling, further regulating liver bile acid metabolism. High expression of small heterodimer partner (Shp) and fibroblast growth factor15 (Fgf15) can cause liver lipid metabolism disorders, whereas MT inhibits the expression of ileal FXR mRNA and reduces the transcriptional levels of the downstream molecules, Shp and Fgf15 [53].

### 4.6. Regulate the Balance Between Day and Night

The normal secretion rhythm of MT in patients with chronic liver diseases is disrupted, which may be related to the impaired clearance of MT due to liver dysfunction. Compared to healthy individuals, patients with liver cirrhosis exhibit significantly higher daytime melatonin levels, with the MT peak occurring later at night [26]. An increase in daytime MT levels can lead to a shift in the biological clock phase and may be associated with sleep disturbances in patients with chronic liver disease [26]. MT reduces the risk of diabetes and cardiovascular diseases by correcting the disrupted biological clock cycle and helps manage metabolic syndromes [54]. Sleep deprivation can lead to weight gain and obesity by increasing food intake and reducing energy expenditure. Studies have found that the expression of peroxisome proliferator-activated receptor γ (PPARγ) in sleep-deprived mice increases, inducing the fat generation process. In a mouse model of sleep deprivation, MT regulates lipid metabolism by activating the AMP-activated protein kinase alpha subunit (AMPKα)/PPARα signaling axis, thereby reducing lipid accumulation in the liver and adipose tissue and preventing weight gain [55]. Sleep deprivation reduces the level of MT, increases the levels of transaminases in plasma, and aggravates the histological abnormalities of the liver tissue of mice with acute liver injury; however, these pathological changes can be reversed by supplementing with MT [56].

**Figure 2:**
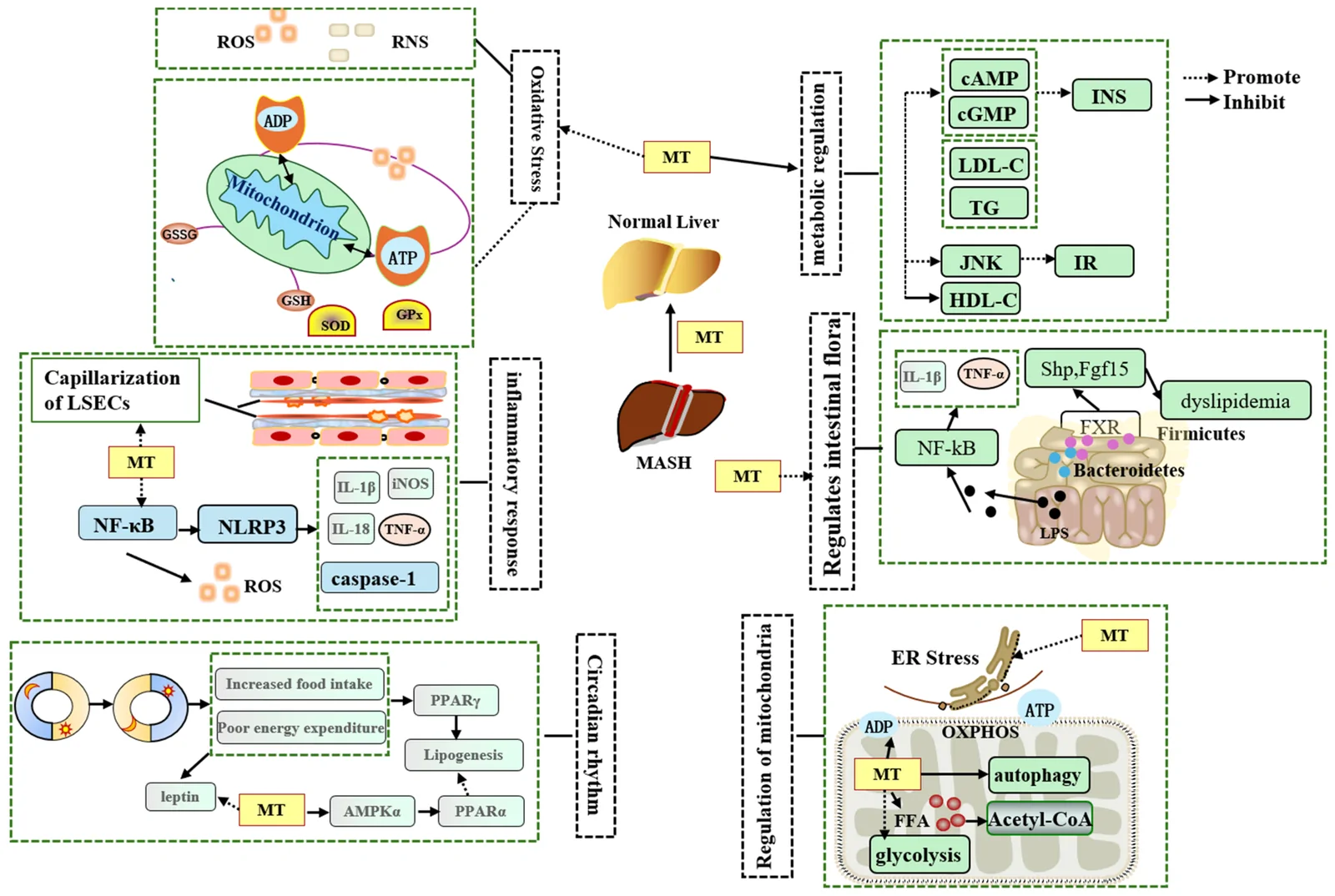
The pathways and mechanisms of MT in MASLD. Note: MT: melatonin; ROS: reactive oxygen species; RNS: reactive nitrogen species; ADP: Adenosine Diphosphate; ATP: adenosine triphosphate; GSSG: oxidized glutathione; GSH: glutathione; SOD: superoxide dismutase; GPx: glutathione peroxidase; LSECs: Liver Sinusoidal Endothelial Cells; NF-κB: nuclear factor kappa-light-chain-enhancer of activated B cells; NLRP3: nucleotide-binding oligomerization domain-like receptor family; IL-1β:Interleukin-1β; IL-18: Interleukin-18; iNOS: inducible nitric oxide synthase; TNF-α:Tumor necrosis factor-α; PPARγ: peroxisome proliferator-activated receptor γ; PPARα: peroxisome proliferator-activated receptor α; AMPKα: Adenosine 5‘-monophosphate-activated protein kinase; cAMP: cyclic adenosine monophosphate; cGMP: cyclic guanosine monophosphate; LDL-C: low-density lipoprotein cholesterol; TG: triglyceride; JNK: c-Jun N-terminal kinase; HDL-C: high-density lipoprotein cholesterol; IR: insulin resistance; INS: insulin; LPS: lipopolysaccharide; FXR: intestinal retinoid X receptor; Shp: small heterodimer partner; Fgf15: fibroblast growth factor 15; ER: Endoplasmic Reticulum; OXPHOS: oxidative phosphorylation; FFA: free fatty acids.

The neuroendocrine feedback system that regulates leptin secretion is tightly controlled by both external food intake and internal circadian rhythm modulators. Abnormal leptin secretion may stem from circadian rhythm disruption [27]. Obesity is characterized by excessive fat accumulation, resulting in elevated levels of leptin in circulation. Serum leptin levels are elevated in patients with NAFLD and are related to NAFLD [57]. Zebrafish exposed to high sugar levels showed increased body weight, obvious liver steatosis, and elevated intestinal leptin levels. After MT administration, liver damage was significantly alleviated, and the expression of intestinal leptin significantly decreased [58]. This further indicates that MT may have a delayed effect on the development of MASLD. The MT action pathways in MASLD are shown in Figure 2.

## 5. Preclinical Research Evidence

Currently, most studies on the effects of MT on MASLD focus on the animal and cellular levels. Male Wistar rats fed a high-fat diet with different doses of MT showed weight loss, a reduced steatosis index, increased oxidative stress, and elevated levels of inflammatory markers. High-dose MT (30 mg/kg) treatment improved hepatic steatosis in rats fed a high-carbohydrate high-fat diet but caused mild hepatotoxicity in healthy rats [59]. In a mouse study, MT significantly ameliorated weight gain, visceral fat accumulation, and hepatic steatosis induced by a high-fat diet. It also suppresses hepatic cholesterol synthesis by downregulating the transcription of SREBP-2 and HMGCR [60]. MT selectively inhibits the levels of proinflammatory CCR3+ monocyte-derived macrophages (MoMFs) in NAFLD mice and upregulates the levels of anti-inflammatory CD206+ MoMFs [61]. It exerts antioxidant effects by reducing oxidative stress [62,63]. However, there is research data indicating that melatonin does not significantly improve hepatic steatosis [64].

Experiments have demonstrated that MT improves pathological hepatic conditions via multiple pathways. It alleviates Cd-induced NAFLD by mitigating mitochondrial damage and oxidative stress, restoring PPAR-α expression, and regulating autophagy function [65]. In a NASH mouse model, MT suppressed lipid accumulation, improved insulin sensitivity, promoted the expression of anti-inflammatory factors, and reduced the severity of liver injury and fibrosis [66]. MT can inhibit inflammasomes through the TLR4/NF-κB pathway, restore normal expression of antioxidant proteins, reduce oxidative protein expression, and mitigate oxidative stress damage [67]. MT suppresses the increase in lipid levels in HepG2 cells induced by fatty acid, down-regulates the expression of PPARα, and enhances β-oxidation or fat breakdown of fatty acids [68]. Mi et al. reported that MT significantly reduced blood glucose levels in ob/ob mice, decreased the level of RBP4 in the liver tissue, reversed macroscopic fatty degeneration of the liver tissue to microfat degeneration, increased the distance between the endoplasmic reticulum and mitochondria, improved the morphology and structure of organelles, and restored the levels of calcium connection protein and ATP synthase [69]. In obese mice, MT not only improves NAFLD by reducing body mass index but also suppresses inflammatory responses by regulating the MAPK-JNK/P38 signaling pathway [70]. In summary, MT can delay the occurrence of MASLD through various pathways such as inhibiting lipid accumulation; improving insulin resistance, antioxidative stress, and anti-inflammation; protecting mitochondrial function; and regulating circadian rhythms. The current studies on MT and MASLD are summarized in Table 1.

There is still controversy surrounding the current research findings. The inconsistencies among study results may be attributed to several factors. First, differences in animal models: studies using high-fat diet models generally observe reduced steatosis, whereas results from studies using methionine-/choline-deficient diet models (which induce more severe steatohepatitis and weight loss) have been inconsistent. Second, differences in MT dose and route of administration may affect its bioavailability. Third, treatment durations have varied, and shorter treatment periods may be insufficient to reverse established steatosis. Fourth, the disease stage at treatment initiation may also influence outcomes; melatonin may be more effective in preventing the progression of steatosis than in reversing advanced disease. Finally, differences in outcome assessment methods (such as histological scoring, biochemical markers, and imaging studies) may also contribute to apparent inconsistencies in results.

Although these findings indicate that MT can reduce the degree of hepatic steatosis and liver fibrosis in experimental models, it should be noted that this evidence comes almost exclusively from rodent studies using high-fat diets or methionine-/choline-deficient diets. Owing to interspecies differences in melatonin receptor distribution and metabolic rate, and because some models lack key comorbidities such as obesity, insulin resistance, and dyslipidemia, extrapolating these results to human MASLD has limitations. In addition, the lack of uniform standards for the dose, route, and timing of MT administration makes comparisons across studies difficult, and the treatment durations are relatively short.

The anti-inflammatory and antioxidant effects of MT have been validated in vitro and in vivo models. However, the MT concentrations used in cell culture experiments often exceed physiological levels by several orders of magnitude, and the in vivo models employed do not fully replicate the chronic low-grade inflammatory state typical of human MASLD. Future preclinical studies should adopt standardized models to improve reproducibility and translational value. Therefore, these preclinical data should be understood as a basis for generating hypotheses rather than as confirmatory evidence.

## 6. Clinical Evidence and Trials

The current clinical evidence regarding the effects of MT on MASLD is insufficient. An increase in serum melatonin levels in patients with NAFLD is correlated with reduced hepatic fibrosis and inflammatory responses. Increased melatonin excretion in the urine is a marker of liver fibrosis [71]. In a study of 74 patients with NAFLD diagnosed through histopathological examination of liver biopsy specimens, when patients with NAFLD were administered MT, their pro-inflammatory cytokine levels decreased significantly, and some lipid metabolism parameters improved [72]. In a few patients with NASH, MT alleviated inflammation in the liver [72]. Another study explored whether long-term maintenance treatment with MT was required in patients with MASLD, and the results demonstrated that MT improved liver enzyme levels in patients with NASH fibrosis. No side effects were observed after long-term treatment (24 weeks). Therefore, longer MT treatment is recommended for patients with NASH [73]; however, long-term use of MT can cause drowsiness, and it is very important to select an appropriate maintenance dose. MT delays the progression of NAFLD and improves liver function-related indicators [74]. One trial showed that MT (12 mg/day) treatment improved some indicators in patients with NAFLD, such as liver enzymes, inflammatory markers, blood pressure, blood glucose, leptin levels, weight, waist circumference, and fatty liver severity [75]. The current clinical studies on MT and MASLD are summarized in Table 2.

The clinical evidence regarding the use of MT in patients with MASLD/MASH remains at a preliminary stage. Most existing studies have relatively small sample sizes, considerable heterogeneity in study design, and inconsistent endpoints across studies. Most studies have relied on surrogate markers, such as changes in serum liver enzymes, inflammatory cytokine levels, or imaging-based assessment of hepatic steatosis, rather than liver biopsy; yet liver biopsy remains the gold standard for evaluating MASH improvement and fibrosis regression. Therefore, further prospective, large-scale, randomized controlled clinical trials are warranted to investigate the association between MT and MASLD.

In the contemporary MASH therapeutic landscape, MT should currently be regarded primarily as an experimental intervention. Although preclinical studies suggest that MT may act on multiple pathogenic pathways relevant to MASH, including oxidative stress, inflammation, mitochondrial dysfunction, and circadian disruption, the available clinical evidence remains preliminary and is not yet sufficient to support its use as a standalone therapy. Moreover, the histological improvements observed in animal models have not been convincingly reproduced in human trials. Therefore, if MT enters clinical practice, it is more likely to serve as an adjunctive therapy alongside lifestyle modification and approved or emerging pharmacological agents, rather than as a primary disease-modifying treatment. Future studies should evaluate melatonin as an add-on intervention to standard-of-care treatment, with rigorous assessment of histological endpoints and careful consideration of patient selection and dosing.

## 7. Conclusions

MASLD is a common metabolic disorder that often leads to hepatic and extra-hepatic complications. The regulatory role of MT in the development of MASLD has garnered significant attention in recent years. As an indole-type hormone, MT functions in regulating the circadian rhythm, maintaining energy balance, preventing oxidative stress, stabilizing mitochondrial structure and function, preventing inflammation, and improving insulin resistance. These functions delay the development of MASLD. Therefore, MT may be regarded as a novel adjunctive therapeutic agent for delaying or inhibiting the onset and progression of MASLD, representing a new direction for the diagnosis and treatment of MASLD.

Current research on MT and MASLD/MASH has largely been conducted at the animal and cellular levels; in the clinical field, more rigorous experiments are needed to clarify the relationship. First, the optimal dose and timing of MT administration in patients with MASLD/MASH have not yet been established. Circadian rhythm disruption is closely linked to the pathogenesis of MASLD, optimizing the timing of administration according to circadian rhythms also warrants further investigation. Second, the drug metabolism and treatment duration of MT in patients with liver disease remain unclear. MASLD/MASH may alter the hepatic metabolism of melatonin, thereby affecting drug exposure and efficacy; therefore, future studies should incorporate pharmacokinetic assessments to guide dose selection. While MT is generally deemed well-tolerated in the short term, its protracted effects on endocrine function, glycemic control, and circadian rhythmicity in patients with chronic liver disease have yet to be adequately elucidated, and more extensive research is warranted in this regard.

In the future, large-scale, multicenter, randomized, placebo-controlled trials with histological endpoints should be conducted. At the same time, surrogate markers such as liver enzymes and imaging-based assessment of steatosis have limited sensitivity for detecting MASH resolution or fibrosis regression, and liver biopsy remains the gold standard. Combination of therapeutic strategies should also be explored, as preclinical studies suggest that MT may enhance the effects of other antioxidants and metabolic modulators.

## Figures and Tables

**Table 1:** Animal models between MT and MASLD/MASH.

Animal Models	Intervention	Effects on the Liver	Ref.
High-fat-diet-induced MASLD C57BL/6 mice	10 and 20 mg/kg, i.p., every other day, for 2 months	MT can delay hepatic steatosis by inhibiting oxidation, reducing lipogenesis, and attenuating lipid peroxidation.	[32]
db/db mice	30 mg/kg/day, i.p., b.i.d., for 8 weeks	MT improved lipid accumulation, fibrosis, and oxidative stress levels.	[37]
High-fat-diet-induced NAFLD rats	10 mg/kg/day, p.o., for 8 weeks	MT reduced the insulin resistance index, liver enzyme activity, and serum total cholesterol and triglyceride levels.	[46]
High-fat-diet-induced MASLD mice	50 mg/kg/day, p.o., for 10 weeks	MT reversed high-fat-diet-induced intestinal dysbiosis and inhibited hepatic steatosis.	[49]
High-carbohydrate high-fat (HCHF) diet-induced NAFLD rats	5, 10, and 30 mg/kg, p.o., for 4 weeks	The 10 mg/kg and 30 mg/kg doses of MT reduced body weight, adiposity index, oxidative damage, and inflammation, but did not affect impaired glucose metabolism. The highest dose (30 mg/kg) reduced the hepatic steatosis index but caused slight liver injury in healthy rats.	[59]
High-fat-diet-induced MASLD mice	50 mg/kg/week, i.p., for 17 weeks	MT may slow the progression of MASLD by reducing intestinal caloric absorption and hepatic cholesterol synthesis.	[60]
Methionine- and choline-deficient (MCD) diet-induced NASH mice	20 mg/kg/day, i.p., for 2 weeks	MT slowed the progression of NASH, reduced hepatic oxidative stress and inflammatory processes, and inhibited DNA damage through its anti-inflammatory and antioxidant effects.	[63]
Methionine- and choline-deficient diet-induced NAFLD pigs	10 mg/kg/day, p.o., for 1 month	MT did not significantly improve hepatic steatosis.	[64]
Methionine- and choline-deficient (MCD) diet-induced NASH mice	20 mg/kg/week, i.p., for 4 weeks	MT inhibited lipid accumulation and peroxidation, improved insulin sensitivity, and attenuated liver inflammation and fibrosis.	[66]
High-Fat-Diet-Induced NASH Model	10 and 20 mg/kg, i.p., every other day, for 6 months	MT protects the liver from inflammation-induced oxidative damage.	[67]
ob/ob mice	100 mg/kg/day, p.o., for 8 weeks	MT inhibited morphological and metabolic liver injury in NAFLD mice.	[69]
High-fat-diet-induced MASLD mice	10 mg/kg/day, p.o., for 12 weeks	MT improves NAFLD by reducing body weight and attenuates inflammation by modulating the MAPK/JNK/p38 signaling pathway.	[70]

Note: NAFLD: non-alcoholic fatty liver disease; MASLD: metabolic dysfunction-associated steatotic liver disease; NASH: nonalcoholic steatohepatitis; MASH: metabolic dysfunction-associated steatohepatitis; MT: melatonin; p.o.: oral administration; i.p.: intraperitoneal injection; MAPK: mitogen-activated protein kinase; JNK: c-Jun N-terminal kinase; p38: p38 mitogen-activated protein kinase.

**Table 2:** Evidence from clinical studies between MT and MASLD/MASH.

Study (Author, Year)	Design	Sample Size	MT Intervention	Duration	Key Index	Main Findings	Limitations
Zemlianitsyna O et al., 2018 [71]	cross-sectional analysis	23 patients with type 2 diabetes mellitus and NAFLD with manifestations of fibrosis	-	-	MT levels in urine	The increase in MT levels in patients with type 2 diabetes mellitus and NAFLD was associated with the presence of fibrotic changes in the liver and a decrease in the activity of the inflammatory process.	Small sample, cross-sectional analysis
Celinski K et al., 2014 [72]	RCT, cohort studies	74 patients with NAFLD diagnosed by liver biopsy (Group I: Essentiale forte and tryptophan; Group II: Essentiale forte and melatonin; Group III: Essentiale forte)	5 mg, b.i.d., p.o.	14 months	GGT, TG, LDL-C, IL-1, IL-6 and TNF-α	MT substantially reduced the levels of pro-inflammatory cytokines and improved some parameters of lipid metabolism in patients with NAFLD.	Small sample
Gonciarz M et al., 2012 [73]	Clinical Trial, a pilot study	42 patients with NASH diagnosed by liver biopsy (30 treated with MT, 12 controls receiving placebo)	5 mg, b.i.d., p.o.	12 weeks	AST, GGT	MT reduced liver enzyme levels in NASH patients.	Small sample, short duration
Cichoz-Lach H et al., 2010 [74]	Controlled Clinical Trial	45 patients with NASH diagnosed by liver biopsy (Group I: Essentiale forte and tryptophan; Group II: Essentiale forte and MT; Group III: Essentiale forte)	5 mg, b.i.d., p.o.	4 weeks	GGT, TG and proinflammatory cytokine levels	MT reduced plasma pro-inflammatory cytokine levels and played a role in the treatment of patients with NASH.	Small sample, short duration
Bahrami M et al., 2020 [75]	RCT, double blind	24 participants in the MT group and 21 participants in the placebo group	12 mg, p.o., once daily at bedtime	12 weeks		MT improved liver enzymes, high-sensitivity C-reactive protein, leptin levels, and fatty liver grades.	Small sample, no liver biopsy

Note: MT, melatonin; NAFLD, non-alcoholic fatty liver disease; NASH, nonalcoholic steatohepatitis; RCT, randomized controlled trial; GGT, gamma-glutamyl transferase; TG, triglyceride; LDL-C, low-density lipoprotein cholesterol; IL-1, interleukin-1; IL-6, interleukin-6; TNF-α, tumor necrosis factor-alpha; p.o.: oral administration; RCT, randomized controlled trial; AST, aspartate aminotransferase; b.i.d., twice daily.

## Data Availability

No new data were created or analyzed in this study. Data sharing is not applicable to this article.
